# Low-Protein Infant Formula Enriched with Alpha-Lactalbumin during Early Infancy May Reduce Insulin Resistance at 12 Months: A Follow-Up of a Randomized Controlled Trial

**DOI:** 10.3390/nu16071026

**Published:** 2024-04-01

**Authors:** Ulrika Tinghäll Nilsson, Bo Lönnerdal, Olle Hernell, Anne Staudt Kvistgaard, Lotte Neergaard Jacobsen, Pia Karlsland Åkeson

**Affiliations:** 1 Department of Clinical Sciences Malmö, Pediatrics, Lund University, 221 00 Lund, Sweden; pia.karlsland_akeson@med.lu.se; 2 Department of Nutrition, University of California, Davis, CA 95616, USA; bllonnerdal@ucdavis.edu; 3 Department of Clinical Sciences, Pediatrics, Umeå University, 901 87 Umeå, Sweden; olle.hernell@umu.se; 4 Arla Foods Ingredients Group P/S, 8260 Viby, Denmark; askv@arlafoods.com (A.S.K.); lojan@arlafoods.com (L.N.J.)

**Keywords:** alpha-lactalbumin, CGMP, infant growth, infant formula, low-protein, infant nutrition, childhood obesity, IGF-1, insulin, body mass index

## Abstract

High protein intake during infancy results in accelerated early weight gain and potentially later obesity. The aim of this follow-up study at 12 months was to evaluate if modified low-protein formulas fed during early infancy have long-term effects on growth and metabolism. In a double-blinded RCT, the ALFoNS study, 245 healthy-term infants received low-protein formulas with either alpha-lactalbumin-enriched whey (α-lac-EW; 1.75 g protein/100 kcal), casein glycomacropeptide-reduced whey (CGMP-RW; 1.76 g protein/100 kcal), or standard infant formula (SF; 2.2 g protein/100 kcal) between 2 and 6 months of age. Breastfed (BF) infants served as a reference. At 12 months, anthropometrics and dietary intake were assessed, and serum was analyzed for insulin, C-peptide, and insulin-like growth factor 1 (IGF-1). Weight gain between 6 and 12 months and BMI at 12 months were higher in the SF than in the BF infants (*p* = 0.019; *p* < 0.001, respectively), but were not significantly different between the low-protein formula groups and the BF group. S-insulin and C-peptide were higher in the SF than in the BF group (*p* < 0.001; *p* = 0.003, respectively), but more alike in the low-protein formula groups and the BF group. Serum IGF-1 at 12 months was similar in all study groups. Conclusion: Feeding modified low-protein formula during early infancy seems to reduce insulin resistance, resulting in more similar growth, serum insulin, and C-peptide concentrations to BF infants at 6-months post intervention. Feeding modified low-protein formula during early infancy results in more similar growth, serum insulin, and C-peptide concentrations to BF infants 6-months post intervention, probably due to reduced insulin resistance in the low-protein groups.

## 1. Introduction

The prevalence of childhood overweight and obesity is increasing. Globally, 37 million children under the age of 5 years were found to be overweight or obese in 2022 [1]. Preventive strategies to reverse this trend are important, both for the individual and from a public health perspective. Systematic reviews indicate that rapid weight gain, especially during the first two years of life, is associated with increased risk of overweight in childhood and later in life [2,3,4,5]. Furthermore, a high protein intake during infancy could result in accelerated early weight gain and increased deposition of fat [6]. According to the early protein hypothesis [7], protein overload results in high serum concentrations of branched-chain amino acids (BCAA), leading to enhanced secretion of insulin and insulin-like growth factor 1 (IGF-1). Previous studies report higher serum insulin [8,9,10,11] and IGF-1 levels [9,10,11] in formula-fed (FF) than in breastfed (BF) infants during the first half of infancy. Thus, the higher protein content in infant formula than in breast milk could contribute to the higher weight gain in FF than in BF infants. Protein concentration in infant formula has been reduced over the past decades and protein quality has improved, based on research by ourselves and by others [12,13,14,15,16,17,18,19,20,21,22,23]. Even though present infant formulas with reduced protein concentration are safe and support age-appropriate growth [24,25,26], they still contain a higher protein concentration than breast milk to supply sufficient amounts of all essential amino acids [27]. Thus, to achieve even lower protein concentration, closer to that of breast milk, changes in the protein composition of infant formulas are needed. We have recently shown appropriate growth up to 6 months in infants fed infant formula with protein content slightly below the EU regulatory lower level, either with alpha-lactalbumin-enriched whey (α-lac-EW) or casein glycomacropeptide-reduced whey (CGMP-RW). Furthermore, weight gain closer to BF infants between 4 and 6 months was found among infants fed α-lac-EW formula. In addition, serum amino acid profiles at 6 months were more like those of BF infants in those fed low-protein formulas compared to infants fed standard (SF) formula [11]. However, does early formula feeding with protein concentrations closer to breast milk induce long-lasting effects on growth? While some studies report that accelerated growth later in childhood is related to the feeding of high-protein infant formula [17,28,29], others find no association [21,30,31]. Systematic reviews thus emphasize the importance of additional follow-up studies to evaluate if reductions in infant formula protein concentration will affect growth and decrease the risk of overweight and obesity in childhood [24,25,26].

The aim of this follow-up study was to evaluate effects on growth and metabolic and hormonal markers, at 12 months, 6 months post-intervention, of feeding low-protein infant formulas with either α-lac-EW or CGMP-RW in relation to standard infant formula or breastmilk in early infancy. Our hypothesis was that growth and metabolic profiles would be more like those of BF infants also 6 months post-intervention in infants fed low-protein formulas during early infancy.

## 2. Materials and Methods

### 2.1. Intervention Study

The ALFoNS study, a double-blinded, controlled, prospective intervention trial was conducted at two centers: the Department of Pediatrics at Skåne University Hospital, Malmö/Lund, and the Department of Pediatrics at University Hospital of Umeå, Sweden. Between December 2014 and November 2019, infants were recruited by invitation letters that were sent to all families with a four-week-old baby in the catchment area. Inclusion criteria were healthy infants, born >37 gestational weeks with birth weight 2500–4500 g, and at least one parent should be able to communicate in Swedish. Exclusion criteria were neonatal problems, malformations, disabilities, or other diseases that could interfere with normal nutrition and growth. Infants born by caesarean section as well as infants that had been treated with antibiotics prior to inclusion were excluded.

In total, 245 exclusively formula-fed infants were randomized to receive either standard infant formula (SF; 2.2 g protein/100 kcal with 10% α-lactalbumin of total protein; *n* = 83) or low-protein infant formulas with either α-lac-EW (1.75 g protein/100 kcal with 27% α-lactalbumin; *n* = 82) or CGMP-RW (1.76 g protein/100 kcal with 14% α-lactalbumin; *n* = 80) from 2 to 6 months of age. Eighty-three exclusively BF infants were enrolled as a reference group, using same inclusion and exclusion criteria and with the mother’s intension to continue exclusively breastfeeding until 6 months of age.

By computer-generated randomization, formula-fed infants were stratified for sex and assigned into random blocks of 6 or 12 to receive SF, α-lac-EW, or CGMP-RW. FF and BF infants were included in a 3:1 sequence with equal numbers of girls and boys. Blinding of the infant formula took place at the formula production site (Laiterie de Montague, Le Planty, France) and the formula were packaged in identical cans that only differed by a code. The group allocation was blinded to families, staff, and investigators. Unblinding, only to investigators, occurred when all infants had attended their 6-month visit and all data and statistical analysis up to 6 months had been performed.

From a pre-study power calculation, a sample size of a minimum 64 infants in each study group was required to detect a difference in weight of 400 g (0.5 SD) at 6 months of age, using a power of 80% and a significant level of *p* < 0.05. To allow a drop-out rate of 20% during intervention, sample size was sized to 80 infants in each study group. For a short period of time, we had a high drop-out rate, and to maintain power an additional 8 infants were included, thus in total 328 infants were included to the ALFoNS study.

Study formulas were provided by Arla Foods Ingredients Group P/S, Denmark and distributed to the families by the study staff. Nutrient and amino acid content of the study infant formulas are presented in Supplementary Table S1. Anthropometry and nutrient intake were assessed monthly from inclusion to 6 months of age, and blood, fecal, and urine samples were obtained at inclusion and at 4 and 6 months. Results on growth and metabolism from the intervention study have recently been reported [11].

### 2.2. Follow-Up Study

Infants who had completed the intervention study (2–6 months of age) were followed up at 12 months of age according to the original ALFoNS study protocol. All infants had completed their 12 months visit in October 2020. The primary outcome of this follow-up study was growth in the period between 6 and 12 months of age and secondary outcomes were serum concentrations of metabolic and hormonal markers (insulin, C-peptide, IGF-1, and leptin) at 12 months of age. This follow-up study was approved by the Regional Ethical Review board at Lund University and performed in accordance with the Declaration of Helsinki. The trial was registered at ClinicalTrials.gov (NCT02410057).

#### 2.2.1. Anthropometrics and Dietary Intake

Anthropometrics were measured; weight recorded with an accuracy of 5 g (Malmö/Lund: UWE AIN 3 or TANITA BD-815MA, Umeå: SECA 757), recumbent length (all sites: SECA 416), and head circumference (HC) (all sites: non-stretchable measuring tape (212 SECA)) with an accuracy of 1 mm. Growth velocity (gains in weight, length, and head circumference) between 6 and 12 months was calculated by dividing the increase in anthropometric measurements by time interval in days between the visits at 6 and 12 month of age. Weight gain is reported as g/d, relative weight gain as g/kg/d, and gain in length and head circumference as mm/d. The study visit was scheduled within a time frame of ±14 days related to the 12-month day; however, a few children had their visit outside this frame due to short-term illness (i.e., fever or milder airway infection). Absolute weight, length, and HC were then extrapolated to reflect the size of the infant at 12 months. Body mass index (BMI) was calculated (kg/m^2^) at 6 and 12 months of age. Z-scores of growth-for-age were calculated relative to standards provided by the WHO [32,33].

After completion of the intervention period at 6 months, all formula-fed infants were switched to common follow-on formulas chosen by the parents. No follow-on study formula was provided from the study and no restrictions regarding nutrient intake were given to the families. The week prior to the 12-month visit, the parents completed a 3-day dietary record where all nutrient intakes were registered in volumes (mL, dL) or weight (g), or as number of tea-, or tablespoons (5 or 15 mL). Breastfeeding was registered for infants still receiving breast milk. Daily nutritional intake was calculated by a dietician using the Dietist Net Pro^®^ database (Kost och Näringsdata AB, Bromma, Sweden), including nutritional data from national and international food agencies and food manufacturers.

#### 2.2.2. Biochemical Analyses

Venous blood samples were collected at least 2 h postprandially. Local anesthetic topical cream was used prior to sampling. The tubes were centrifuged for 10 min at 1300× *g*. Serum was separated into Cryo tubes and then stored at −80 °C at the Biobank in Lund or Umeå until analysis. Hemoglobin (Hb) was analyzed directly at the University and Regional Laboratories of Skåne or Norrland’s University Hospital with the Sysmex XN-10 (Sysmex Corporation, Chuoko, Japan). The tubes were transported from the Biobanks frozen on dry ice to the respective laboratory for analysis. Serum (s) insulin, C-peptide, and IGF-1 were analyzed at the University and Regional Laboratories of Skåne, insulin and C-peptide with the Cobas 601 instrument (Roche Diagnostics, Rotkreuz, Switzerland) and IGF-1 by the IDS-iSYS assay (Immunodiagnostic System Ltd., Boldon, Tyne & Wear, UK). Serum leptin and leptin receptor were analyzed by ELISA (Human Leptin ELISA kit, EMD Millipore; Merck KGaA, Darmstadt, Germany, and Human Leptin R Quantikine^®^ ELISA, R&D Systems Inc., Minneapolis, MN, USA) at the Pediatric Research Laboratory at Umeå University.

#### 2.2.3. Statistical Analyses

IBM SPSS Statistics version 28.0 (Released 2021. IBM SPSS Statistics for Windows, Version 28.0. Armonk, NY, USA: IBM Corp.) was used for all statistical calculations. For group comparison of normally distributed continuous data, one way-analysis of variance (ANOVA) with post-hoc Bonferroni test was used. Separate one-way ANOVA with post-hoc Bonferroni test for the three FF groups was also performed. Results are presented as mean ± SD. Non-normally distributed continuous data were logarithmically transformed (natural logarithms) before group comparison and results presented as geometric mean and confidence interval. Furthermore, adjusted analysis was performed for growth data with one-way ANCOVA post hoc Bonferroni test, where adjustments were made for baseline value of the specific outcome (if applicable), maternal weight gain and smoking during pregnancy, gestational diabetes, maternal and paternal BMI. Growth data were additionally analyzed separately for the group of infants that completed the intervention study with full adherence to the study protocol (i.e., the PP population in the intervention study). Multiple linear regression was used for assessment of associations between metabolic and hormonal markers (insulin, C-peptide, IGF-1, and leptin) and anthropometric data at 12 months and growth rate between 6 and 12 months of age, and adjustments were made for baseline value and feeding group. Significance level was set at *p* < 0.05 for all analyses.

## 3. Results

### 3.1. Study Groups

**Figure 1:**
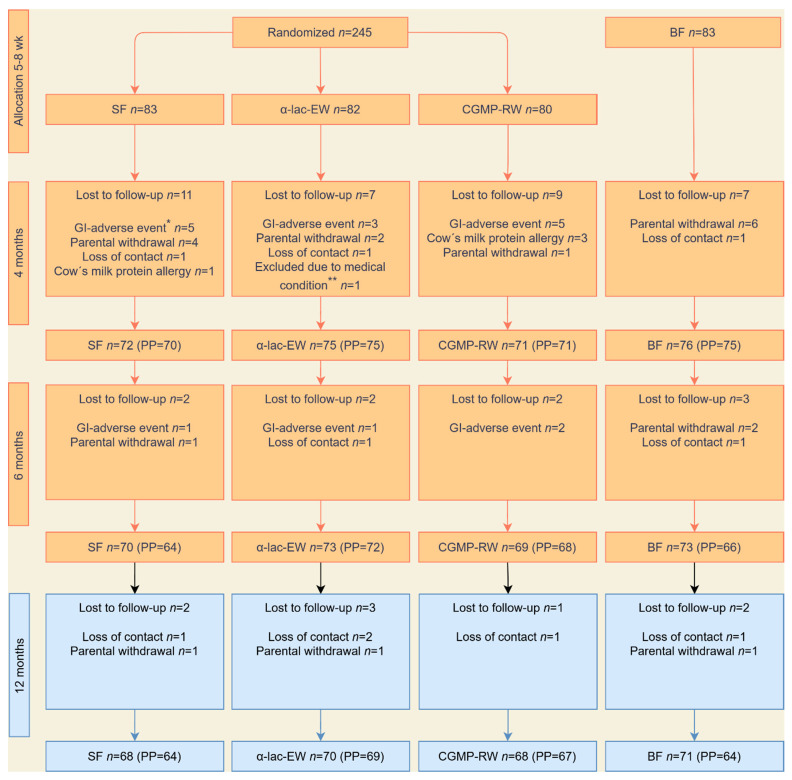
Study flowchart for the intervention period and the follow-up at 12 months. Randomization, allocation, intervention, and follow-up in intention-to-treat (ITT) and per protocol population (PP). SF, standard formula; α-lac-EW, experimental formula with α-lactalbumin-enriched whey; CGMP-RW, experimental formula with CGMP-reduced whey; BF, breastfed. Reasons for lost to follow-up: * Gastrointestinal adverse events, such as vomiting, stomach ache, flatulence, or constipation. ** Congenital genetic disease affecting growth.

Of the 328 infants included in the intervention study, 285 completed the intervention up to 6 months of age (SF = 70, α-lac-EW = 73, CGMP-RW = 69 and BF = 73), of whom 277 infants (SF = 68, α-lac-EW = 70, CGMP-RW = 68 and BF = 71) participated in the follow-up visits at 12 month of age (Figure 1). Drop-out rate between 6 and 12 months was low, with no difference between the groups.

Characteristics of the follow-up infant population and their parents are presented in Table 1.

### 3.2. Growth

Weight gain between 6 and 12 months (g/d) was similar in the low-protein formula groups and the BF group, but lower than in the SF group (SF vs. α-lac-EW; *p* = 0.022, SF vs. CGMP-RW; *p* = 0.003 and SF vs. BF; *p* = 0.019) (Table 2). At 12 months, mean weight and BMI did not differ between the formula groups. Mean weight was not significantly different in the α-lac-EW and BF groups (*p* = 0.05) at this age, but it was slightly higher in the α-lac-EW than in the BF-group when adjusting for weight at inclusion (Table 2). BMI at 12 months in infants in the low-protein formula groups were more comparable to the BF group, whereas infants in the SF group had higher BMI than the BF group (*p* < 0.001). Relative weight gain between 6 and 12 months, expressed as g/kg/d, was lower in the CGMP-RW group than in the SF group, *p* = 0.002. All results persisted in the adjusted analyses.

**Figure 2:**
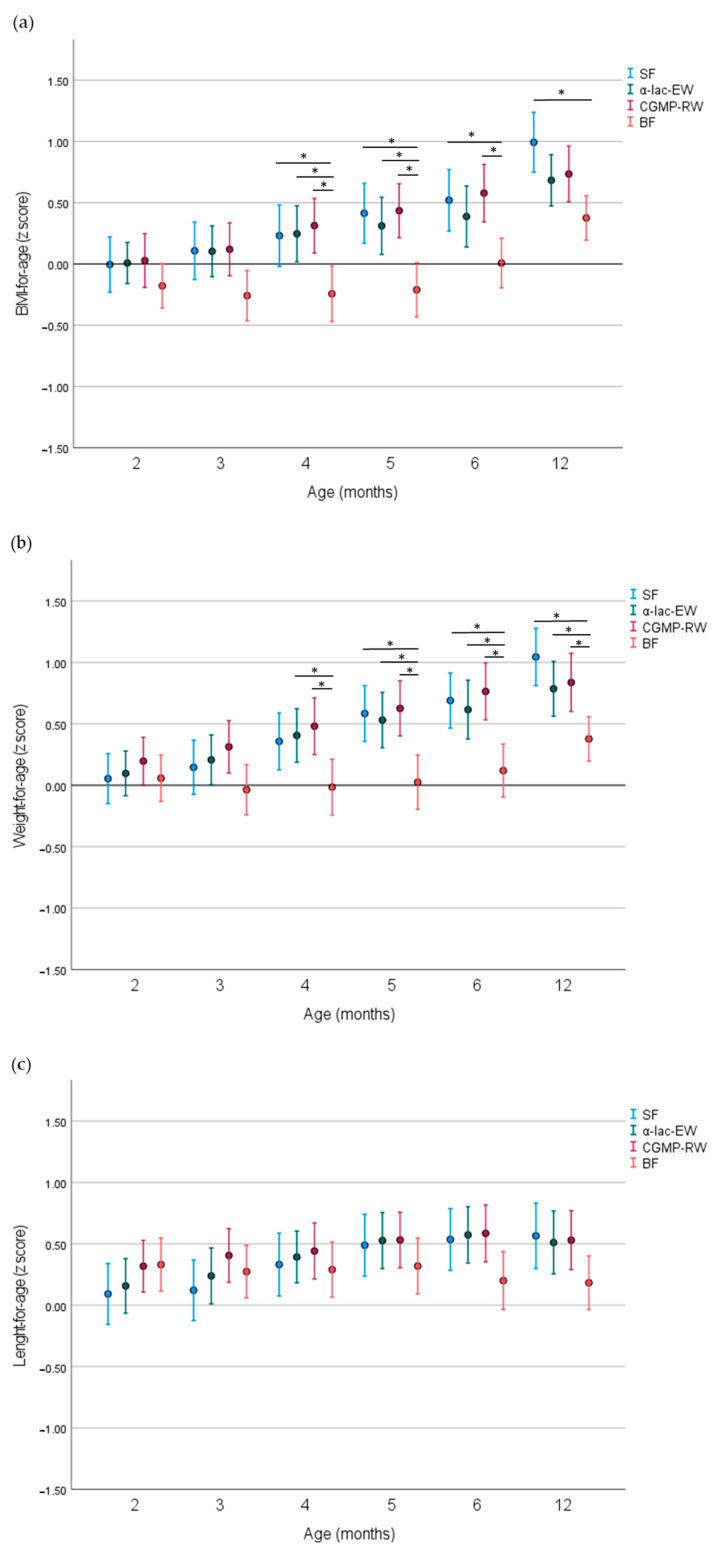
(**a**–**c**) Unadjusted mean (95% CI) z-score for BMI-for-age, weight-for-age, and length-for-age in study groups. SF, standard formula; α-lac-EW, experimental formula with α-lactalbumin-enriched whey; CGMP-RW, experimental formula with CGMP-reduced whey; BF, breastfed. Groups compared by one-way ANOVA, post-hoc Bonferroni. * *p* value < 0.05.

At 12 months, BMI z-score was more comparable among both low-protein formula groups and BF infants but was higher in the SF compared to the BF group (*p* < 0.001), (Table 2, Figure 2a). Weight-for-age z-scores (WAZ) were similar and higher in all FF groups compared to the BF group (Table 2, Figure 2b), weight-for-length z score (WLZ) were similar in all FF groups but was higher in the SF than in the BF group (Table 2). Length-for-age z-score (LAZ) (Table 2, Figure 2c) and head circumference-for-age z-score (HCZ) were similar in all study groups at 12 months (Table 2). In the adjusted analysis, LAZ was higher in all FF groups compared to the BF group (Table 2).

In infants who completed the intervention study with full adherence to the study protocol, mean weight at 12 months was not significantly different between low-protein formula groups and the BF group, whereas other growth data in this subpopulation did not differ from the results in the total follow-up population (Supplementary Table S2).

At 12 months, 37% of the infants in the BF group received breast milk to some extent. However, BMI at 12 months and weight gain between 6 and 12 months did not differ between infants still breastfed at 12 months and those who were not, but mean weight was slightly lower among infants still breastfed at 12 months, *p* = 0.047 (Supplementary Table S3).

### 3.3. Nutrient Intake

Data on dietary intake of energy and macronutrients (protein, fat, and carbohydrates) at 12 months are presented in Table 3. No significant differences in nutrient intake were found among the study groups. Twenty-six out of 71 infants in the BF group were still partly breastfed at 12 months of age. Since the amount of breast milk was not measured, their total nutrient intake could not be calculated, and these children have not been included in the analyses of nutritional data. Dietary records for four FF children and for one BF child were incomplete and could thus not be used.

### 3.4. Biochemistry

Biochemical parameters are presented in Table 4. At 12 months, S-insulin and C-peptide concentrations were more similar in both the low-protein formula and BF groups, whereas infants in the SF group had higher serum insulin (*p* < 0.001) and C-peptide (*p* = 0.003) than the BF group, and higher serum C-peptide than the CGMP-RW group (*p* = 0.044). Serum IGF-1 concentrations at 12 months were similar in all groups, as were S-leptin, soluble leptin receptor (SLR), free leptin index (leptin/SLR), and hemoglobin concentrations. Data on these parameters at 4 and 6 months of age in infants participating in the follow-up study at 12 months are presented in Supplementary Table S4.

### 3.5. Associations between Hormonal and Metabolic Markers and Growth

Serum insulin and IGF-1 at 4 and 6 months of age were positively associated with mean weight and length at 12 months. S-IGF-1 at 12 months was positively associated with mean weight, length, and BMI at 12 months and with weight gain between 6 and 12 months. S-insulin at 12 months was positively associated with mean length at 12 months and with weight gain between 6 and 12 months (Table 5). Serum C-peptide at 4 months was positively associated with mean weight at 12 months, and at 4 and 12 months it was positively associated with mean length at 12 months (Table 5).

During intervention, as well as at follow-up, S-leptin was positively associated with mean weight and BMI, and at 6 and 12 months it was positively associated with mean length. At 12 months, S-leptin was positively associated with weight gain between 6 and 12 months, but at 4 months it was negatively associated with the same parameter (Table 5).

## 4. Discussion

Our findings that growth as well as serum insulin and C-peptide at follow-up at 12 months of age were more similar to BF infants among infants fed low-protein infant formula with either α-lac-EW or CGMP-RW during early infancy show that low protein intake, closer to that of BF infants, also affects growth 6 months post-intervention, probably through lower insulin resistance.

High protein intake from infant formula has been linked to enhanced growth during infancy, but also to increased weight in childhood. In the CHOP study, where infants were fed a formula with very-high protein concentrations (2.9 g/100 kcal between 0 and 4 months and 4.4 g/100 kcal between 4 and 12 months), BMI at 2 years as well as mean weight up to 2 years were higher in the high-protein compared to the low-protein formula group (1.77 g/100 kcal from 0–4 months and 2.2 g/100 kcal from 4–12 months) and the BF group [17]. Higher BMI was also found at 6 years [29] and at 11 years post-intervention among infants fed the high-protein formula [28]. In contrast, with moderately elevated formula protein concentrations, as in the EPOCH study (2.7 g protein/100 kcal vs. 1.8 g protein/100 kcal) [9], and in the BeMIM study (2.2 g protein/100 kcal vs. 1.89 g/100 kcal) [16], anthropometric parameters did not differ significantly between the previously FF and BF infants at follow-up from 9 months to 5 years of age [9] or at 4 years [31] or 7 years [30], respectively.

The conflicting results from these short- and long-term follow-up studies may partly be explained by differences in protein intake during infancy, but they also underline the need for more follow-ups of RCTs evaluating whether lower protein formulas with modified protein composition in early infancy may reduce the risk of overweight and obesity during childhood.

We recently found higher serum insulin and C-peptide at 6 months by the end of intervention in all formula groups compared to BF infants [11], and these results are in line with previous findings [9]. In contrast, in the present follow-up at 12 months, S-insulin and C-peptide concentrations were slightly lower in the low-protein formula groups compared to the SF group and were more similar to the BF group. Our results suggest that a higher protein intake in early infancy, as in the SF group, results in higher insulin resistance at 12 months compared to the low-protein formula groups and the BF group. These new findings are in contrast to results in the EPOCH study, where serum insulin and C-peptide 5 months post-intervention were similar in all formula groups and the BF group [9].

Elevated serum branched-chain amino acid (BCAAs) concentrations have been suggested to precede insulin resistance and to be associated with the development of overweight and obesity [34,35,36]. Even though S-BCAAs were higher in all formula groups compared to the BF group during the intervention, S-BCAAs were lower in the low-protein formula groups than in the SF group at 6 months [11]. Furthermore, at this age, weight gain and BMI were more similar in the low-protein formula groups and the BF group [11]. Thus, it is possible that the BCAA concentrations did influence the insulin concentration post-intervention, resulting in lower weight gain (g/d) between 6 and 12 months and lower BMI at 12 months in the low-protein formula groups compared to the SF group, resulting in growth rates closer to BF infants.

Furthermore, in the present study, serum insulin at 12 months, but not at 6 months, was associated with weight gain (g/d) between 6 and 12 months. Our results suggest potential imprinting of insulin secretion by protein intake during the intervention period. Previous follow-up studies found no associations between insulin concentrations at the end of intervention and growth post-intervention up to 2 years in the study by Kouwenhoven [8], or at 7 years of age in the BeMIM study [30]. However, in contrast to our study, in those studies blood sampling was not performed post-intervention.

The importance of insulin and C-peptide concentrations for long-term growth is emphasized by results from a prospective cohort study from the Netherlands [37]. In this study, elevated insulin and C-peptide concentrations at the age of 6 years were associated with accelerated weight, length, and BMI from infancy and up to 6 years of age, which could result from increased insulin resistance. The authors suggested increased body fat mass to be the mechanism for the higher insulin concentration. However, since nutrient intake was not assessed in the Dutch study, the role of protein intake during infancy could not be evaluated. In the CHOP cohort, higher body fat mass up to 6 years of age was found in the high-protein formula group [38], whereas no differences in body composition were found in the EPOCH [9] or in the BeMIM studies [30,31], where protein concentration in the “high-protein” formula was only moderately elevated. It is unknown if our findings of higher serum insulin and C-peptide, and higher growth parameters at 6 months post-intervention in the SF group compared to the low-protein groups, will persist later in childhood.

S-IGF-1 has been found to be higher in FF compared to BF infants during the first half of infancy by ourselves and by others [9,10,11,39,40]. However, in the present study, S-IGF-1 at 12 months did not differ between the FF groups and the BF group, which contrasts with findings in the EPOCH study [9], where S-IGF-1 was higher in FF compared to BF infants 5 months post-intervention. S-IGF-1 has also been found to be positively associated with mean weight during the first year of life [10,39,40], as in our study, and with BMI [39,40]. In addition, an association between S-IGF-1 at 4 months and long-term growth 7 years post-intervention was recently reported [30]. However, other studies report no association with IGF-1 in early infancy and growth during the first years of life [8,41]. In the Cambridge Baby study, S-IGF-1 at 3 months was only associated with length gain, but not with weight gain up to 12 months, suggesting that other factors could be important for the accelerated weight gain seen in FF infants [39].

Accelerated weight gain during childhood, particularly during the first two years of life, has been linked to the development of auto-antibodies to pancreatic beta-cells, a first sign of the autoimmune process leading to clinical type 1 diabetes in genetically predisposed children [42,43]. Furthermore, associations have recently been found between high protein-energy intake in early childhood and increased risk of developing a specific type of auto-antibody to pancreatic beta-cells (GAD65) in children at risk of developing type 1 diabetes [44]. The precise mechanism behind this association is yet to be clarified, but protein-induced high BCAA concentrations with subsequently high insulin and IGF-1 levels are likely to be involved in the process. These findings raise the question of whether or not feeding a low-protein infant formula may reduce the development of GAD65- autoantibodies in infants at risk for developing type 1 diabetes, a hypothesis to be followed up by subsequent studies.

Concerns about the potential long-term consequences of high-protein formula intake and increased risk of kidney disease have been raised. Feeding infants formula with very-high protein content has been shown to increase kidney volume and function in healthy infants at 6 months of age [45], an effect probably mediated by IGF-1 [46]. If combined with overweight during childhood, the ensuing kidney hypertrophy may not be reversible, resulting in impaired kidney function and increased risk of hypertension, as pointed out by the authors [46].

Leptin, as an appetite-regulating hormone, is important for the adjustment of food intake, energy balance, and metabolism. In the present study, S-leptin at 6 and 12 months was found to be associated with mean weight and BMI at both time points, and at 12 months with length and weight gain between 6 and 12 months. A long-term association of s-leptin at 4 months and growth was also found by Kouwenhoven et al. [8]. However, despite differences in protein intake between the groups during the intervention period, S-leptin was similar in all study groups at 6 [11] and at 12 months in the present study. Thus, our data indicate that the influence of leptin on growth is mediated by factors other than protein intake.

The soluble leptin receptor (SLR) is the main binding protein for leptin in humans and modulates its activity [47]. It has been suggested that SLR is present in breast milk, where it might influence the infant’s appetite and satiety by regulating the availability of leptin [48]. In the present study, S-SLR at 12 months was found to be lower in infants still BF compared to those no longer BF in the BF group (Supplementary Table S3). In addition, within the BF group, mean weight at 12 months was slightly lower in those who were still BF compared to infants who were no longer BF (Supplementary Table S3). Whether these findings are related to the lower S-SLR in infants still BF remains to be further investigated.

Our findings 6 months post-intervention of the potential effects of different protein intakes during early infancy are interesting, since early-life nutrition may impact the metabolic response in the infant through metabolic imprinting, thus modifying the risk of developing metabolic disorders such as obesity [49,50]. Thus, lower protein intake during early infancy, through imprinting effects on serum insulin and C-peptide, may result in growth parameters more comparable to BF infants also 6 months post-intervention. Our data suggest that feeding a protein-reduced infant formula with the addition of α-lac-EW or CGMP-RW in early infancy induces positive lasting metabolic effects.

Growth and metabolic parameters did not differ substantially in the present study between the two different low-protein formula groups, with either α-lac-EW or CGMP-RW, both with increased concentration of α-lac, 27% and 14%, respectively, compared to the SF formula with 10% α-lac (Supplementary Table S1). This suggests that the modified protein quality by either approach, together with a reduction in formula protein concentration, slightly below the lower limit of the EU recommendations, represents two possible alternatives of lowering protein in infant formula.

Our overall aim in this study was to evaluate possible imprinting on growth and certain metabolic markers of feeding low-protein formula during the first 6 months of life. In the present study, after the intervention period, no study follow-on formula was provided so the child was fed according to the parents’ choice. If there was an imprinting effect of what was fed during the first 6 months, this would be revealed at follow-up, regardless of what had been fed between 6 and 12 months.

Continued partial breastfeeding up to 2 years of age is recommended by the WHO and UNICEF [51]. In our study population, 37% of the infants in the BF group were still partly BF at 12 months of age, a higher proportion than among Swedish infants in general [52]. Since breastmilk intake was not measured, total nutrient intake could not be calculated for these infants. However, BMI and weight gain (Supplementary Table S3), as well as serum insulin, C-peptide, and IGF-1 concentrations (Supplementary Table S3) at 12 months did not differ within the BF group among infants who were still BF compared to those who were not, although infants still BF weighed slightly less.

A strength of our study is that metabolic and hormonal markers (IGF-1, insulin, C-peptide, leptin), were analyzed, not only during intervention, but also 6 months post-intervention, which enables us to better evaluate whether low-protein formula given during the first half of infancy influence growth and the metabolic profile 6 months post-intervention. Further, almost all infants (97%) that completed the intervention period participated in this follow-up study and 84% of infants originally included in the study groups remained in the study at follow-up.

A limitation of the study is that body composition was not measured and thus the proportions between fat mass and fat-free mass could not be evaluated.

## 5. Conclusions

In conclusion, feeding a modified low-protein infant formula early in life results in more similar growth 6 months post-intervention to that of BF infants compared to feeding a standard formula with higher protein concentration, which probably resulted from reduced insulin resistance in the low-protein formula groups. Hence, a further reduction of formula protein concentration by modifying protein quality as in the present study may be a strategy for early prevention of childhood overweight and obesity. Continued follow-up of the study population is thus desirable to evaluate whether our findings persist later in childhood.

## Figures and Tables

**Table 1:** Characteristics of infants and parents in the follow-up population.

	SF *n* = 68	α-lac-EW *n* = 70	CGMP-RW *n* = 68	BF *n* = 71
Gestational age (week)	39.6 ± 1.2	39.7 ± 1.3	40.1 ± 1.2	40.0 ± 1.1
Birth weight (g)	3498 ± 460	3519 ± 450	3590 ± 455	3552 ± 432
Birth length (cm)	50.4 ± 2.2	50.2 ± 2.0	50.4 ± 1.7	50.4 ± 1.9
Birth head circumference (cm)	34.7 ± 1.2	35.0 ± 1.3	35.0 ± 1.2	35.1 ± 1.5
Age at inclusion (d)	49.6 ± 5.0	49.4 ± 4.4	49.2 ± 5.9	50.5 ± 4.7
Ever breastfed before inclusion (*n* (%))	50 (72)	55 (79)	60 (88)	71 (100)
Days of breast-feeding before inclusion (*n*)	14.9 ± 14.2	17.9 ± 15.3	18.2 ± 14.2	50.0 ± 4.8
Male (*n* (%))	35 (51)	34 (49)	34 (50)	33 (47)
Complementary feeding during intervention (*n* (%)) ^1^	22 (32)	25 (37)	26 (39)	22 (32)
Age at follow-up visit (m)	12.0 ± 0.23	12.0 ± 0.32	12.0 ± 0.36	12.1 ± 0.34
Maternal age (y)	31.4 ± 4.8	31.6 ± 4.5	31.6 ± 4.4	32.6 ± 4.3
Maternal origin (*n* (%))				
Nordic	66 (96)	64 (91)	64 (94)	64 (90)
European (non-Nordic)	2 (3)	3 (4)	3 (4)	5 (7)
Non-European	1 (1)	3 (4)	1 (1)	2 (3)
Maternal higher education (*n* (%)) ^2^	37 (54)	42 (60)	49 (72)	57 (80)
Maternal smoking during pregnancy (*n* (%))	3 (4)	3 (4)	2 (3)	0 (0)
Maternal BMI at enrollment (kg/m^2^)	28.4 ± 5.2	27.3 ± 5.1	26.3 ± 4.3	25.1 ± 3.6
Weight gain during pregnancy (kg)	12.8 ± 6.4	13.6 ± 6.8	14.9 ± 6.3	14.0 ± 5.3
Gestational diabetes (*n* (%))	2 (3)	1 (1)	4 (6)	5 (7)
Paternal origin (*n* (%))				
Nordic	57 (85)	63 (93)	63(94)	55 (80)
European (non-Nordic)	4 (6)	3 (4)	2 (3)	9 (13)
Non-European	6 (9)	2(3)	2 (3)	5 (7)
Paternal higher education (*n* (%))	25 (37)	30 (44)	40 (60)	50 (72)
Paternal smoking during pregnancy (*n* (%))	11 (16)	6 (9)	3 (5)	7 (10)
Paternal BMI (kg/m^2^)	26.6 ± 4.1	27.1 ± 5.8	26.4 ± 4.0	25.8 ± 3.6

SF, standard formula; α-lac-EW, experimental formula with α-lactalbumin-enriched whey; CGMP-RW, experimental formula with CGMP-reduced whey; BF, breastfed. Data presented as mean ± SD or as number (%). ^1^ Intake of ≥2 tsp per day at 6 months of age. ^2^ University or higher professional education.

**Table 2:** Anthropometric data * at 6 and 12 months and growth velocity between 6 and 12 months in all study groups.

		SF		α-lac-EW		CGMP-RW	*p*-Value ^1,2^		BF
	*n*		*n*		*n*			*n*	
Weight (g)									
6 mo	70	8323 ± 879 ^a^	73	8228 ± 1025 ^a^	69	8342 ± 1009 ^a^	0.76 (0.67)	73	7771 ± 923
12 mo	68	10,584 ± 1222 ^a^	70	10,236 ± 1205	68	10,315 ± 1214 ^a^	0.22 (0.11)	71	9719 ± 962
Weight gain									
6–12 mo (g/d)	68	12.5 ± 3.5 ^a,b,c^	70	11.0 ± 2.7	68	10.7 ± 3.2	0.002 (0.008)	70 ^#^	10.9 ± 2.9
6–12 mo (g/kg/d)	68	1.2 ± 0.23 ^c^	70	1.1 ± 0.23	68	1.0 ± 0.25	0.002 (0.008)	70 ^#^	1.1 ± 0.27
Length (cm)									
6 mo	70	67.9 ± 2.5	73	67.9 ± 2.4	69	67.8 ± 2.3	0.99 (0.98)	73	67.1 ± 2.6
12 mo	68	76.3 ± 2.8	70	76.1 ± 2.9	68	76.2 ± 2.4	0.91 (0.19)	71	75.2 ± 2.4
Length gain (mm/d)									
6–12 mo	68	0.46 ± 0.09	70	0.45 ± 0.07	68	0.45 ± 0.06	0.64 (0.70)	70 ^#^	0.45 ± 0.06
HC ** (cm)									
6 mo	70	43.9 ± 1.2	73	43.9 ± 1.6	69	43.9 ± 1.6	0.84 (0.71)	73	43.7 ± 1.5
12 mo	68	46.8 ± 1.2	70	46.7 ± 1.5	68	46.7 ± 1.7	0.90 (0.077)	71	46.5 ± 1.4
HC gain (mm/d)									
6–12 mo	68	0.2 ± 0.03	70	0.2 ± 0.03	68	0.2 ± 0.03	0.21 (0.18)	70 ^#^	0.2 ± 0.03
BMI (kg/m^2^)									
6 mo	70	18.1 ± 1.5 ^a^	73	17.8 ± 1.6	69	18.1 ± 1.5 ^a^	0.51 (0.44)	73	17.2 ± 1.3
12 mo	68	18.2 ± 1.6 ^a^	70	17.6 ± 1.4	68	17.7 ± 1.5	0.081(0.11)	71	17.1 ± 1.1
*Z*-score 12 mo									
BMIZ	68	0.99 ± 1.01 ^a^	70	0.68 ± 0.88	68	0.74 ± 0.94	0.12 (0.16)	71	0.38 ± 0.75
WAZ	68	1.04 ± 0.96 ^a^	70	0.79 ± 0.94 ^a^	68	0.84 ± 0.98 ^a^	0.25 (0.07)	71	0.37 ± 0.75
WLZ	68	1.07 ± 0.99 ^a^	70	0.76 ± 0.89	68	0.81 ± 0.96	0.13 (0.28)	71	0.40 ± 0.75
LAZ	68	0.56 ± 1.10	70	0.51 ± 1.07	68	0.53 ± 0.99	0.95 (0.28)	71	0.17 ± 0.91
HCZ	68	0.97 ± 0.87	70	0.92 ± 1.01	68	0.91 ± 1.11	0.94 (0.56)	71	0.80 ± 0.94

* Unadjusted values. SF, standard formula; α-lac-EW, experimental formula with α-lactalbumin-enriched whey; CGMP-RW, experimental formula with CGMP-reduced whey; BF, breastfed. Mo, months. Data presented as mean ± SD. ** HC = Head circumferences. WAZ, weight-for-age z-score; WLZ, weight-for-length z-score; LAZ, length-for-age z-score; HCZ, head circumference-for-age z-score; BMIZ, BMI-for-age. ^#^ One infant, 3 months late for the 6 months visit, was excluded from weight, length, and HC gain analysis between 6–12 months. ^1^ Formula groups compared by one-way ANOVA, post-hoc Bonferroni. ^2^ Formula groups compared by one-way ANCOVA, post-hoc Bonferroni, adjusted for baseline value of the specific outcome (if applicable), weight gain during pregnancy, gestational diabetes, maternal smoking during pregnancy, maternal and paternal BMI. ^a^ Significantly different vs. BF (*p* < 0.05). ^b^ SF vs. α-lac-EW. ^c^ SF vs. CGMP-RW.

**Table 3:** Daily nutrient intake at 12 months of age in all study groups calculated from 3-day food diary.

	SF *n* = 66	α-lac-EW *n* = 68	CGMP-RW *n* = 68	*p*-Value ^1^	BF *n* = 44 ^#^
Energy (kcal)	891 ± 170	892 ± 174	866 ± 157	0.61	866 ±183
Energy (kcal/kg)	85.4 ± 18.8	87.7 ± 15.9	84.6 ± 15.6	0.55	87.7 ± 17.9
Protein (g)	28.6 ± 7.0	30.4 ± 8.0	28.8 ±7.0	0.30	29.2 ±7.0
Protein (g/kg)	2.7 ± 0.6	3.0 ± 0.8	2.8 ± 0.7	0.083	3.0 ± 0.7
Fat (g)	34.1 ± 8.4	35.0 ± 10.1	33.3 ± 8.2	0.56	33.1 ± 10.1
Fat (g/kg)	3.3 ± 0.9	3.4 ± 1.0	3.3 ± 0.8	0.44	3.4 ± 1.0
Carbohydrate (g)	111.2 ± 21.1	108.0 ± 22.8	106.8 ± 20.3	0.48	107.1 ± 25.0
Carbohydrate (g/kg)	10.7 ± 2.3	10.6 ± 2.0	10.4 ± 1.9	0.78	10.8 ± 2.2

SF, standard formula; α-lac-EW, experimental formula with α-lactalbumin-enriched whey; CGMP-RW, experimental formula with CGMP-reduced whey; BF, breastfed. Data presented as mean ± SD ^1^ Formula groups compared by one-way ANOVA, post-hoc Bonferroni. ^#^ 26 children partly breastfed were excluded from the analyses.

**Table 4:** Serum insulin, C-peptide, IGF-1, leptin, soluble leptin receptor (SLR), and hemoglobin (Hb) at 12 months of age in study groups.

		SF		α-lac-EW		CGMP-RW	*p*-Value ^1,2^		BF
	*n*		*n*		*n*			*n*	
Insulin (mIU/L)	64	6.07 (4.96–7.43) ^a^	67	4.78 (3.94–5.78)	65	4.31 (3.47–5.36)	0.056 (0.092)	63	3.28 (2.56–4.19)
C-peptide (nmol/L)	64	0.73 ± 0.37 ^a,b^	67	0.62 ± 0.36	65	0.59 ± 0.24	0.035 (0.046)	63	0.53 ± 0.28
IGF-1 (µg/L)	64	67.8 (62.2–73.9)	67	61.6 (55.6–68.3)	65	63.3 (57.5–69.7)	0.35 (0.86)	63	59.5 (53.7–65.9)
Leptin (ng/mL)	62	2.81 (2.33–3.40)	65	2.22 (1.86–2.65)	63	2.49 (2.16–2.87)	0.15 (0.062)	57	2.42 (2.07–2.83)
SLR (ng/mL)	63	44.2 ± 7.9	65	43.3 ± 10.9	64	44.1 ± 10.5	0.85 (0.86)	58	45.1 ± 9.8
Hb (g/L)	58	118 ± 10	62	119 ± 7	60	119 ± 7	0.72 (0.53)	56	116 ± 8

SF, standard formula; α-lac-EW, experimental formula with α-lactalbumin-enriched whey; CGMP-RW, experimental formula with CGMP-reduced whey; BF, breastfed. Data presented as mean ± SD or geometric mean (95% CI), unadjusted values. ^1^ Formula groups compared by one-way ANOVA, post- hoc Bonferroni. ^2^ Formula groups compared by one-way ANCOVA, post-hoc Bonferroni adjusted for outcome variable at baseline, weight gain during pregnancy, gestational diabetes, maternal smoking during pregnancy, maternal and paternal BMI. ^a^ Significantly different vs. BF (*p* < 0.05). ^b^ SF vs. CGMP-RW.

**Table 5:** Associations between hormonal and metabolic markers at 4, 6, and 12 months and growth outcome from 6 to 12 months of age in study population.

Metabolic and Hormonal Marker	Age (mo)	Weight (g)			Length (cm)			BMI			Weight Gain 6–12 mo (g/d)		
		Β	CI (95%)	*p*	Β	CI (95%)	*p*	Β	CI (95%)	*p*	B	CI (95%)	*p*
Insulin 4 mo													
	6	348	212, 484	<0.001	0.6	0.3, 0.9	<0.001	0.5	0.2, 0.7	<0.001			
	12	369	183, 555	<0.001	0.9	0.5, 1.3	<0.001	0.3	0.01, 0.5	0.27	0.09	−0.5, 0.8	0.70
Insulin 6 mo													
	6	280	146, 414	<0.001	0.4	0.08, 0.7	0.014	0.4	0.15, 0.6	0.001			
	12	205	415, 395	0.035	0.6	0.2, 1.0	0.002	0.06	−0.2, 0.3	0.65	−0.4	−1.0, 0.2	0.19
Insulin 12 mo													
	12	130	−19, 279	0.086	0.4	0.06, 0.7	0.021	0.03	−0.2, 0.2	0.78	0.7	0.2, 1.1	0.005
C-peptide 4 mo													
	6	776	356, 1197	<0.001	0.8	−0.2, 1.8	0.11	1.2	0.5, 2.0	0.001			
	12	792	222, 1362	0.007	1.5	0.2, 2.7	0.027	0.8	−0.03, 1.5	0.06	−0.03	−1.8, 1.8	0.97
C-peptide 6 mo													
	6	760	330, 1190	<0.001	0.9	−0.07, 2.0	0.067	1.1	0.4, 1.9	0.004			
	12	576	−30, 1181	0.063	1.3	0.02, 2.6	0.047	0.4	−0.4, 1.1	0.32	−0.9	−2.8, 1.1	0.37
C-peptide 12 mo													
	12	166	−23, 557	0.41	0.01	−0.8, 0.9	0.023	0.2	−0.3, 0.7	0.39	1.1	−0.1, 2.4	0.073
IGF-1 4 mo													
	6	798	471, 1126	<0.001	1.7	1.0, 2.4	<0.001	0.8	0.3, 1.4	0.005			
	12	829	385, 1272	<0.001	2.4	1.5, 3.3	<0.001	0.3	−0.3, 0.9	0.33	0.2	−1.3, 1.6	0.83
IGF-1 6 mo													
	6	587	317, 857	<0.001	0.9	0.3, 1.5	0.002	0.8	0.3, 1.3	0.001			
	12	640	259, 1022	0.001	2.3	1.5, 3.0	<0.001	0.04	−0.5, 0.5	0.88	0.4	−0.9, 1.6	0.55
IGF-1 12 mo													
	12	945	626, 1264	<0.001	2.1	1.5, 2.8	<0.001	0.6	0.2, 1.0	0.006	3.0	2.0, 4.0	<0.001
Leptin 4 mo													
	6	570	400, 738	<0.001	0.1	−0.3, 0.6	0.57	1.2	0.9, 1.4	<0.001			
	12	422	182, 663	<0.001	0.2	−0.4, 0.8	0.48	0.6	0.3, 0.9	<0.001	−0.9	−1.6, 0.09	0.028
Leptin 6 mo													
	6	644	472, 815	<0.001	0.2	−0.3, 0.6	0.41	1.3	1.0, 1.6	<0.001			
	12	704	451, 958	<0.001	0.6	0.01, 1.1	0.046	1.0	0.7, 1.3	<0.001	0.3	−0.6, 1.2	0.50
Leptin 12 mo													
	12	793	605, 982	<0.001	0.6	0.5, 1.4	<0.001	0.9	0.7, 1.2	<0.001	2.0	1.4, 2.6	<0.001

Multiple linear regression analyses were used for the assessment of associations between metabolic and hormonal markers and anthropometric data at 6 and 12 months and growth rate between 6 and 12 months of age, and adjustments were made for baseline value and feeding group. Bcoefficients. Mo, months.

## Data Availability

The data presented in this study are available on request from the corresponding author due to privacy of research participants.

## Supplementary Materials

The following supporting information can be downloaded at: https://www.mdpi.com/article/10.3390/nu16071026/s1, Table S1: Energy, nutrient, and amino acid composition of study infant formulas and minimum levels according to EU regulation; Table S2: Anthropometric data at 6 and 12 months and growth velocity between 6 and 12 months of age in infants that completed the intervention study with full adherence to the study protocol; Table S3: Anthropometric data at 12 months, weight gain between 6 and 12 months, and biochemistry data in the BF group, with (BM^+^) or without (BM^-^) breast milk intake at 12 months of age; Table S4: Serum insulin, C-peptide, IGF-1, and leptin at 4 and 6 months in infants that participated in follow-up study at 12 months of age. Reference [53] are cited in the supplementary materials
