# Supplementary material for: Low-Protein Infant Formula Enriched with Alpha-Lactalbumin during Early Infancy May Reduce Insulin Resistance at 12 Months: A Follow-Up of a Randomized Controlled Trial
Source: Nutrients. 2024 Apr 1;16(7):1026. doi: 10.3390/nu16071026 (PMC11013926; doi:10.3390/nu16071026)
Supplement: Supplementary file 1 [file nutrients-16-01026-s001.zip › nutrients-2916567-supplementary.pdf]

Supplement material

Low Protein Infant Formula Enriched with Alpha-Lactalbumin during Early Infancy May Reduce Insulin Resistance at 12 Months: A Follow-Up of a Randomized Controlled Trial

Ulrika Tinghäll Nilsson, Bo Lönnerdal, Olle Hernell, Lotte Neergaard Jacobsen,
Anne Staudt Kvistgaard, Pia Karlsland Åkeson

Table S1. Energy-, nutrient-, and amino acid composition of study infant formulas and minimum levels according to EU regulation.

	**SF [1]**	**α-lac-EW**	**CGMP-RW**	**EU-Regulation [2]**
Energy (kcal/100 mL)	67.3	68.2	68.0	60
Whey: casein ratio	60:40	70:30	70:30	
Protein (g/100 kcal)	2.20	1.75	1.76	1.8
Protein (g/100 mL)	1.48	1.19	1.20	
α-lactalbumin (%)	10	27	14	
Fat (g/100 kcal)	5.6	5.7	5.7	4.4
Carbohydrate (g/100 kcal)	10.1	10.1	10.2	9
Sodium (mg/100 kcal)	27.0	27.0	29.0	20
Potassium (mg/100 kcal)	96.8	91.9	91.0	60
Calcium (mg/100 kcal)	102.0	100.0	98.0	50
Chloride (mg/100 kcal)	70.4	71.0	69.0	50
Iodine (µg/100 kcal)	35.9	32.9	34.0	10
Phosphorous (mg/100 kcal)	57.1	63.6	64.1	25
Selenium (µg/100 kcal)	5.5	5.0	5.1	1
Magnesium (mg/100 kcal)	10.0	10.0	10.0	5
Zinc (mg/100 kcal)	1.2	1.2	1.2	0.5
Iron (mg/100 kcal)	1.2	1.2	1.1	0.3
Copper (µg/100 kcal)	67.1	69.0	59.7	35
Manganese (µg/100 kcal)	54.6	53.6	53.1	1
Vitamin A (µg ER/100 kcal)	163.2	171.0	166.7	60
Thiamine (B1) (µg/100 kcal	257.9	253.5	252.2	60
Riboflavin (B2) (µg/100 kcal)	345.1	314.9	324.2	80
Niacin (B3) (µg/100 kcal)	1257.8	1270.0	1299.0	300
Pantothenic acid (B5) (µg/100 kcal)	1338.3	1256.0	1298.0	400
Pyridoxin (B6) (µg/100 kcal)	153.4	157.0	153.6	35
Biotin (B8) (µg/100 kcal)	6.3	5.9	6.0	1.5
Folic acid (B9) (µg/100 kcal)	44.9	41.6	42.0	10
Cobalamin (B12) (µg/100 kcal)	0.45	0.31	0.37	0.1
Vitamin C (mg/100 kcal)	23.0	23.8	23.5	10
Vitamin D3 (µg/100 kcal)	2.2	2.3	2.3	1
Vitamin E (mg αTE/100 kcal)	4.2	4.2	4.4	0.5
Vitamin K (µg/100 kcal)	19.4	20.6	20.8	4
Essential amino acids (mg/100 kcal)				
Cysteine	37	39	39	38
Histidine	56	42	45	40
Isoleucine	127	111	96	90
Leucine	225	160	201	166
Lysine	208	162	177	113
Methionine	53	36	42	23

Phenylalanine	89	65	72	83
Threonine	127	117	87	77
Tryptophan	37	36	35	32
Tyrosine	82	56	66	76
Valine	135	103	98	88
Nonessential amino acids (mg/100 kcal)				
Alanine	93	60	77	-
Arginine	65	45	51	-
Aspartic acids	211	186	182	-
Glutamic acid	439	314	344	-
Glycine	44	36	36	-
Proline	177	118	115	-
Serine	114	90	85	-

[1] SF; standard formula, α-lac-EW; experimental formula with α-lactalbumin-enriched whey, CGMP-RW; experimental formula with CGMP-reduced whey. [2] EU Regulation [53].

Table S2. Anthropometric data * at 6 and 12 months and growth velocity between 6 and 12 months of age in infants that completed the intervention study with full adherence to the study protocol.

		**SF**		**α-lac-EW**		**CGMP-RW**	***p*-Value [1(2)]**		**BF**
	n		N		n			n	
Weight (g)									
6 mo	66	8306 ± 823 [2,a]	72	8235 ± 1031	68	8337 ± 1015 [a]	0.82 (0.75)	69	7829 ± 940
12 mo	64	10,544 ± 1074 [a]	69	10,257 ± 1202	67	10,297 ± 1214	0.31 (0.093)	64	9801 ± 962
Weight gain									
6-12 mo (g/d)	64	12.3 ± 2.7 [a,b,c]	69	11.1 ± 2.6	67	10.6 ± 3.1	0.002 (0.005)	64	11.0 ± 3.0
6-12 mo (g/kg/d)	64	1.16 ± 0.20 [c]	69	1.08 ± 0.23	67	1.02 ± 0.25	0.002 (0.005)	64	1.12 ± 0.28
Length (cm)									
6 mo	66	68.0 ± 2.5	72	68.0 ± 2.4	68	67.9 ± 2.3	0.27 (0.99)	66	67.2 ± 2.6
12 mo	64	76.3 ± 2.7	69	76.1 ± 2.9	67	76.2 ± 2.4	0.94 (0.079)	64	75.4 ± 2.5
Length gain (mm/d)									
6-12 mo	64	0.46 ± 0.09	69	0.45 ± 0.07	67	0.45 ± 0.06	0.81 (0.62)	64	0.45 ± 0.06
HC ** (cm)									
6 mo	66	43.9 ± 1.2	72	43.8 ± 1.6	68	43.8 ± 1.5	0.87 (0.63)	66	43.7 ± 1.5
12 mo	64	46.8 ± 1.2	69	46.7 ± 1.6	67	46.6 ± 1.6	0.74 (0.85)	64	46.5 v 1.4
BMI (kg/m^2)									
6 mon	66	18.0 ± 1.4 [a]	72	17.8 ± 1.6	68	18.1 ± 1.6 [a]	0.65 (0.63)	66	17.3 ± 1.3
12 mon	64	18.1 ± 1.5 [a]	69	17.7 ± 1.3	67	17.7 ± 1.5	0.15 (0.17)	64	17.2 ± 1.1
Z score 12 mo									
BMIZ	64	0.96 ± 0.97 [a]	69	0.71 ± 0.86	67	0.72 ± 0.94	0.23 (0.39)	64	0.42 ± 0.76
WAZ	64	1.02 ± 0.87 [a]	69	0.81 ± 0.92	67	0.82 ± 0.98	0.36 (0.41)	64	0.42 ± 0.77
WLZ	64	1.03 ± 0.93 [a]	69	0.79 ± 0.87	67	0.80 ± 0.98	0.23 (0.35)	64	0.45 ± 0.75
LAZ	64	0.56 ± 1.06	69	0.52 ± 1.08	67	0.52 ± 1.00	0.96 (0.89)	64	0.20 ± 0.94
HCZ	64	0.98 ± 0.88	69	0.93± 1.02	67	0.87 + 1.07	0.83 (0.74)	64	0.81 ± 0.93

* Unadjusted values. SF; standard formula, α-lac-EW; experimental formula with α-lactalbumin-enriched whey, CGMP-RW; experimental formula with CGMP-reduced whey, BF; breastfed. Data presented as mean ± SD. ** HC = Head circumferences. WAZ; weight-for-age z-score, WLZ; weight-for-length z-score, LAZ; length-for-age z-score, HCZ; head circumference-for-age z-score, BMIZ; BMI-for-age. [1] Formula groups compared by one-way ANOVA, post-hoc Bonferroni. [2] Formula groups compared by one-way ANCOVA, post-hoc Bonferroni, adjusted for baseline value of the specific outcome (if applicable), weight gain during pregnancy, gestational diabetes,

maternal smoking during pregnancy, maternal and paternal BMI. [a] Significantly different vs. BF (p< 0.05). [b] SF vs. α-lac-EW. [c] SF vs. CGMP-RW.

Table S3. Anthropometric data at 12 months, weight gain between 6 to 12 months and biochemistry data in the BF group, with (BM+) or without (BM-) breastmilk intake at 12 months of age.

		BM+		BM-	*p*-Value [1]
	n		n		
Mean weight	26	7706 ± 841	45	8076 ± 991	0.042
BMI	26 [#]	16.9 ± 1.1	45	17.3 ± 1.1	0.19
Weight gain (g/d)	25	10.2 ± 2.8	45	11.2 ± 3.0	0.20
Weight gain (g/kg/day)	25	1.90 ± 0.10	45	2.10 ± 0.09	0.061
IGF-1 (μg/L)	22	58.56 (47.29; 52.51)	41	60.02 (54.44; 67.41)	0.82
Insulin (mIU/L)	22	2.43 (1.62; 3.63)	41	3.85 (2.82; 5.26)	0.075
C-peptide (nmol/L)	22	0.45 ± 0.26	41	0.57 ± 0.28	0.1
Leptin (ng/ml)	19	2.70 (2.14; 3.41)	38	2.30 (1.87; 2.83)	0.33
SLR (ng/ml)	19	40.2 ± 7.26	39	47.38 ± 10.16	0.009
Hb (g/l)	19	115.4 ± 6.9	37	115.6 ± 8.1	0.91

SLR = soluble leptin receptor. [1] Independent-Samples T-test. Data presented as mean ± SD or as geometric mean (95% CI) [#] One infant was 3 months late for 6 months visit, excluded from weight gain analysis between 6-12 months.

Table S4. Serum insulin, C-peptide, IGF-1 and leptin at 4 and 6 months in infants that participated in follow-up study at 12 months of age.

		SF		α-lac-EW		CGMP-RW	*p*-Value [1(2)]		BF
	n		n		n			n	
Insulin (mIU/L)									
4 mo	64	8.6 ± 5.1 [a]	67	7.8 ± 4.7 [a]	64	8.9 ± 6.1 [a]	0.62 (0.63)	64	4.9 ± 2.5
6 mo	66	6.8 ± 4.4 [a]	66	6.7 ± 5.2 [a]	64	7.1 ± 4.7 [a]	0.61 (0.56)	66	4.0 ± 2.7
C-peptide (nmol/L)									
4 mo	64	0.62 ± 0.20 [a]	67	0.61 ± 0.23 [a]	64	0.64 ± 0.23 [a]	0.88 (0.75)	64	0.43 ± 0.16
6 mo	66	0.54 ± 0.19 [a]	66	0.53 ± 0.22 [a]	64	0.54 ± 0.22 [a]	0.98 (0.95)	66	0.37 ± 0.17
IGF-1 (μg/L)									
4 mo	64	70.9 ± 22.6 [a]	67	70.7 ± 20.5 [a]	64	69.1 ± 22.6 [a]	0.88 (0.76)	64	55.0 ± 19.2
6 mo	66	60.2 ± 20.2 [a]	66	62.3 ± 23.1 [a]	64	61.0 ± 18.8 [a]	0.84 (0.96)	66	44.7 ± 15.4
Leptin (ng/ml)									
4 mo	58	5.2 ± 2.8	55	6.1 ± 3.8	59	6.3 ± 3.5	0.15 (0.065)	55	6.7 ± 4.4
6 mo	60	4.8 ± 2.6	61	5.4 ± 3.7	69	5.2 ± 2.7	0.56 (0.47)	56	4.7 ± 2.7

SF, standard formula; α-lac-EW, experimental formula with α-lactalbumin-enriched whey; CGMP-RW, experimental formula with reduced CGMP whey; BF, breastfed. Data presented as mean ± SD, unadjusted values. [1] Formula groups compared by one-way ANOVA, post- hoc Bonferroni. [2] Formula groups compared by one-way ANCOVA, post-hoc Bonferroni, adjusted for weight gain during pregnancy, gestational diabetes, maternal smoking during pregnancy, maternal and paternal BMI. [a] Significantly different vs. BF (p <0.05).
